# WWOX-Related Neurodevelopmental Disorders: Models and Future Perspectives

**DOI:** 10.3390/cells10113082

**Published:** 2021-11-09

**Authors:** Daniel J. Steinberg, Rami I. Aqeilan

**Affiliations:** The Concern Foundation Laboratories, The Lautenberg Center for Immunology and Cancer Research, Department of Immunology and Cancer Research-IMRIC, Faculty of Medicine, The Hebrew University of Jerusalem, Jerusalem 91120, Israel; Daniel.steinberg@mail.huji.ac.il

**Keywords:** WOREE syndrome, SCAR12, brain organoids, knockout, models

## Abstract

The WW domain-containing oxidoreductase (*WWOX*) gene was originally discovered as a putative tumor suppressor spanning the common fragile site FRA16D, but as time has progressed the extent of its pleiotropic function has become apparent. At present, WWOX is a major source of interest in the context of neurological disorders, and more specifically developmental and epileptic encephalopathies (DEEs). This review article aims to introduce the many model systems used through the years to study its function and roles in neuropathies. Similarities and fundamental differences between rodent and human models are discussed. Finally, future perspectives and promising research avenues are suggested.

## 1. A Brief Introduction to WWOX in the Central Nervous System

The goal of the vast field of neuroscience is to enhance our understanding of the complex apparatus and mechanisms composing the nervous system [1]. Two approaches to achieve this, which are not mutually exclusive, are the study of its normal development and function and the study of “when it goes wrong”, i.e., the study of neuropathology.

The WW domain-containing oxidoreductase (*WWOX*) gene is gaining increasing attention for its possible role in both normal central nervous system (CNS) development, and its involvement in neurological diseases [2,3,4,5,6,7,8,9]. The *WWOX* gene was first discovered in the early 2000s due to its colocalization in the chromosomal common fragile site (CFS) FRA16D associated with cancer [10], which in turn led to thorough investigations of its role in tumor suppression, DNA damage response, metabolism, and cellular homeostasis, among other functions [11,12,13,14,15]. A murine ortholog was also cloned which allowed its study in rodent models [16].

The human *WWOX* gene spans 1.1 Mb but due to its large introns, translates into a 2.2kb mRNA and a 1.245kb ORF encoding a 46 kDa protein composed of 414 amino acids [10,17]. The protein contains two N-terminal WW domains (referred to as WW1 and WW2) and a catalytic domain in the C-terminus, which is homologous to short-chain dehydrogenase/reductase (SDR) family proteins [10,16,18], although its substrate is currently unknown [19]. These domains allow WWOX to interact with many partner proteins, effectively functioning as an adaptor protein and as a transcriptional repressor, all of which are reviewed elsewhere [12,20,21,22].

Although the WWOX protein can be found in many tissues, it is highly expressed in the brain, with variable expression during different stages of embryogenesis and postpartum maturation [3,8,23,24]. This has led to a working hypothesis that WWOX has a central role in CNS development and homeostasis and, by extension, that its loss can lead to neurological disorders. This review aims to discuss the different models made available through the years to study *WWOX*-related developmental and epileptic disorders and possible treatment avenues that became reachable through these models. It is, however, crucial to understand that *WWOX* has been associated with many other disorders such as autism-spectrum disorders (ASD), Alzheimer’s disease (AD), Parkinson’s disease (PD), and multiple sclerosis (MS), the models of which will be briefly reviewed. 

## 2. Rodent Models of WWOX Loss-of-Function

### 2.1. Rats

In late 2007, Suzuki et al. published a rat model that presented with severe dwarfism, early postnatal lethality, and high incidence of seizures (characterized as wild running that progressed to generalized tonic-clonic epileptic seizures), and was therefore termed *Ide/Ide* rats (lethal dwarfism with epilepsy). This model was derived through experimental inbreeding that resulted in a spontaneous mutation in an unknown gene, which in 2009 was revealed to be a 13 bp deletion in exon 9 of the *Wwox* gene. The mutations were inherited in an autosomal recessive manner and severely decreased the WWOX protein expression in the hippocampus [25,26].

The affected rats demonstrated growth retardation beyond the age of 3 days postnatally, a relative increase in the brain weight (approximately 5.2 ± 1.4% of the body weight in the *Ide/Ide* rats compared to 2.5 ± 0.25% in normal rats), ataxic gait (95% of the studied rats), and audiogenic epileptic seizures (34% spontaneously, 95% following sound stimulation via a cage speaker), with early lethality starting by the age of 21 days (100% of the affected rats died between the ages of 77–84 days). Electroencephalography (EEG) recordings revealed low wave complexes at low-frequency ranges (5–6 Hz) during clonic convulsions, and sporadic interictal spikes (at about 10 Hz), which were greater in the occipital than in the frontal electrodes. Histologically, extracellular vacuoles were present in the amygdala and hippocampus of affected rats [25,26]. Interestingly, analysis of the rats’ testis revealed decreased WWOX expression, accompanied by testicular size abnormalities, increased apoptosis of germ cells, lack of spermatocytes, and immature Leydig cells [25,26,27]. Additionally, biochemical abnormalities were found in the analyzed blood, including increased concentrations of plasma urea nitrogen, creatinine, and inorganic phosphate, as well as decreased concentrations of plasma growth hormone [25]. At this time point, rats were also used to study the role of WWOX in sciatic nerve transection [28], in a study that found *Wwox* to be a key determinant in the crossroad when “choosing” between neuronal survival and death. This could occur through interactions with important transcription factors found *in-vitro*, such as CREB, AP-1, c-JUN, Elk-1, and NF-kB. Later on, its role was expanded in the study of traumatic brain injury, showing increased complex formation of WWOX/Hyal-2 and accumulation in the apoptotic nuclei of neurons [29]. This supported *in-vitro* data that showed WWOX acting as a bridge for Hyal-2 and Smad4 interaction and promoting bubbling cell death (BCD).

Further research into the *Ide/Ide* rats came approximately 10 years later, with the Suzuki group performing immunobiological analysis of both wild-type and mutated rodents. The group reported that under normal conditions, at day 21 post-natal, WWOX expression could be detected in the cerebral cortex, hippocampus, corpus callosum (CC), diencephalon (thalamus, hypothalamus, habenular nuclei), internal capsule, cerebellum, brain stem, spinal cord, and olfactory bulb. Interestingly, the expression was mainly apparent in the layers II-III and V of the wild-type rodent cortex and the white matter [30]. Examination of *Ide/Ide* rats led to several interesting observations: first, WWOX expression was diminished in all the sites mentioned above, and second, WWOX mutations led to several changes in cardinal cellular populations in the brain. From the neuronal standpoint, although unchanged in numbers, the neurons (marked by neuronal specific nuclear protein NeuN) had reduced neurite growth, which was attributed to abnormal neuronal differentiation. As for the glial cells, the myelin content (marked by MBP and CNP) and the number of oligodendrocytes (marked by APC) were found to be reduced in the cortex and CC, together with significantly lower numbers of astrocytes (marked by GFAP) and microglia (marked by IBA-1) [30]. The latter is of particular interest for the well-documented astrogliosis and microglial activations that usually accompany seizures [31,32,33,34,35,36,37]. Furthermore, despite the reduction in the number of neurites, glial cells, and myelination, the group reported no change in cortical thickness [30].

Next, Iacomino et al., (2020) examined *lde/lde* rats at postnatal day one and found a reduced number of foliations in the cerebellum, and impaired neuronal migration of late-born neurons in the cortical plate, and defected cortical layers formation [38].

The comprehensive work on *Ide/Ide* rats has substantially increased our knowledge of WWOX in the rodent CNS, but at the time of writing this review, lacked the mechanistic dissection of its role. More light in this matter was shed by the rat’s distant cousin—the mouse.

### 2.2. Mice

The first indication for the role of WWOX in the mouse brain came in early 2004 by the Chang group, who reported differential expression in different brain areas during the stages from development from E9 up to adulthood [24]. For example, in the telencephalon and its structures, WWOX was detected as early as E12, with relative peak levels seen in the adult cortex. In contrast, in the diencephalon, WWOX expression was reproducibly detected only around E16-E18 at low-moderate levels, and in the choroid plexus, it was detected at E14 with gradually increasing levels until adulthood. 

Aldaz and Hussain reviewed mRNA data from publicly accessible mouse in-situ hybridization (ISH) and RNA sequencing (RNA-seq) results [8]. Briefly, data from the Allen Mouse Brain Atlas found high *Wwox* expression in the dorsal zone of layer II of the medial entorhinal cortex (a region in the hippocampal formation), and in the anterior and posterior regions of the basolateral amygdalar nuclei in the cortical subplate. *Wwox* was expressed to a much lesser extent in the somatosensory and somatomotor cortices, and the cerebellar cortex. An RNA-seq dataset from postnatal day 7 (P7) mice showed uniform expression in neurons and all glial cell types, with abundant expression in oligodendrocyte progenitor cells (OPCs) and in mature myelinating oligodendrocytes (mature OLs). An important note is the stable expression of *Wwox* in microglia cells, which is upregulated upon lipopolysaccharide (LPS) injection and induction of inflammation. 

**Figure 1:**
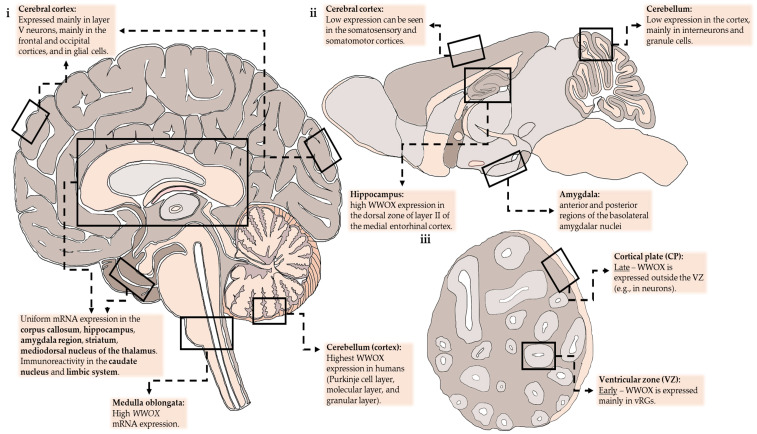
Anatomical regions with a moderate-high expression of WWOX demonstrated schematically in sections of the adult human brain (**i**), adult mice brain (**ii**), and brain organoids (**iii**). vRGs—ventricular radial glia.

At the single-cell level, when analyzing single-cell RNA-seq (scRNA-seq) data from the DropViz database (http://dropviz.org/), *Wwox* expression seemed to vary between neuronal subtypes and regions (in the cerebellum for instance), and *Wwox* was expressed most in interneurons and granule cells. In the cortex, it is most seen in deep-layer pyramidal neurons (specifically from layer V). In the hippocampus, *Wwox*-expressing neurons were found in the medial entorhinal cortex. Other non-neuronal cell types that express high *Wwox* transcripts were ependymal cells and choroid plexus cells. *Wwox* expression in the mouse brain is summarized in Figure 1. 

It was not until 2007 that Aqeilan et al. introduced the first rodent model for *Wwox* mutations (as the *Ide/Ide* rats were only associated with WWOX in 2009) in the form of the *Wwox*-null mice [11]. This highly anticipated model, which is sometimes referred to as the “conventional null mice” was obtained by targeted disruption of a 6 kb section of the *Wwox* genomic sequence, including exons 2,3, and 4. Briefly, a targeting cassette containing in-frame sequences for the *LacZ* and *Neo* genes and two genotyping probes, flanked by homology arms, were electroporated into mouse embryonic stem cells (mESCs). The result was congenic B6-129 mixed genetic background mice harboring the disrupted gene, which could then be bred with the genotype of the born pups following classic mendelian ratios. As reported in the original paper and follow-up publications, the mice suffered from growth retardation, alterations in serum levels of proteins, carbohydrates, calcium, and lipids, osteopenia, bone metabolic defects, high tumor burden, and eventually from postnatal lethality [39]. Similar to rats, low levels of WWOX were also associated with impaired steroidogenesis and gonadal abnormalities [40,41].

These phenotypes were validated in a second generation of *Wwox*-null mice, referred to here as conditional null mice, reported by Ludes-Meyers et al., [42]. The researchers further observed bone impairment, growth retardation, metabolic and hematologic defects, and a short lifespan. Here the approach was different. The use of the Cre-LoxP system in C57Bl/6 background mice resulted in a flanked exon 1 and Cre-recombinase expressed under the general *EllA* promotor. A similar approach was taken, and validated the recapitulation of the original null phenotype in a conditional knock-out (KO) system, by Abdeen et al., [43]. This model has allowed for vast research underlining the role of *WWOX* in many tissues and different types of cancer, some of which can be found in [44,45,46,47].

Another noteworthy model for *WWOX* mutations in mice is the *Wwox* hypomorphic mice [41], a model that was published soon after the conventional null mice. In these animals, the WWOX transcript levels were attenuated using gene-trap technology that was inserted into exon 4 of mESCs clone XG218. This manipulation generated a *Wwox* splice variant containing exons 1–4 spliced in-frame with the *β*-geo gene, a WWOX protein lacking the SDR domain and, overall, a 95% reduction in *Wwox* mRNA levels in the homozygous mice and concordant reduction in protein levels in most, but not all, tissues. These mice are fundamentally different from the null mice, both in the gene expression, but also in the observed phenotypes. Although testicular defects were observed, the above-mentioned bone abnormalities were not. Furthermore, although lifespan was reduced compared to the control mice, it was significantly prolonged compared to the null mice, and these mice were reported to suffer from a higher burden of B-cell lymphomas (only in females). Interestingly, no neurological-related phenotypes were reported in the *Wwox* hypomorphic mouse model, suggesting the minute expression of WWOX in the brain, which was not assessed [41], could be enough to maintain normal CNS function.

Despite the extensive aforementioned research, the next advancement in the neuroscientific research of *WWOX* came in late 2014 when, among other important discoveries expanded upon later, the first association between *Wwox* mutations and CNS abnormalities in mice was published [48]. The researchers recapitulated the epileptic phenotypes described in *Ide/Ide* rats in the conditional null mice and reported both spontaneous and audiogenic seizures. The convulsions started around 2-weeks of age postnatally and were described as wild running and jumping, progressing to tonic-clonic movements of the limbs and tail, followed by a lethargic post-ictal stage. KO mice also presented with balance disturbances, implying an ataxic phenotype.

About four years later, this mouse strain was subjected to deeper investigations [49]. First, the inhibitory interneurons population of the hippocampus was examined, which showed a significant reduction in parvalbumin (PV) expressing neurons in the dentate gyrus (DG) and CA1 regions. There was an additional 30% reduction in Neuropeptide Y (NPY) expressing GABAergic interneurons that was limited to the DG. In concordance with this, quantification of the glutamate decarboxylase isoforms GAD65/67 showed a marked reduction as measured by immunoblot analysis. Interestingly, in contrast to the rat-derived data, the hippocampus showed signs of neuroinflammation, as observed by mild microgliosis (IBA-1 staining in CA1, CA3, and entire hippocampus) and astrogliosis (GFAP staining in CA1, CA3, and entire hippocampus). Furthermore, the researchers isolated neural stem cell (NSC) neurospheres from WT and *Wwox*-KO hippocampi and performed bulk RNA-seq. Comparative transcriptomic analysis demonstrated 283 differentially expressed genes (log2 FC > ± 1, *p*-value < 0.005, FDR < 0.05), of which 184 transcripts were up-regulated and 99 were down-regulated in KO samples. Ingenuity Pathway Analysis (IPA) identified the dysregulated genes to be related to ‘nervous system development and function’, ‘neurological disease’, ‘epilepsy’, ‘seizure disorder’, ‘cognitive impairment’ and ‘neurodegeneration’. Genes were identified that were implicated in epilepsy and validated them using RT-PCR in the whole hippocampus.

In 2020, Cheng et al. published two new strains of *Wwox*-KO mice using the Cre-LoxP system in mESCs before blastocyst injection. One mimicked the previously published genomic editing in conditional null mice by targeting exon 1, and the other the conventional null mice by targeting exons 2-4 [50]. First, they assessed cerebellar function. Although the test did not find any significant differences between WT and heterozygous mice, the null mice suffered from gait ataxia and severe impairment in motor coordination, grip strength, and balance. The cerebellum itself in the null mice had fused vermian lobules VI and VII, foliation defects in lobules V, VI, and VII, and partial loss of the Purkinje cells with diminished expression of calbindin around postnatal day 20. A TUNEL assay showed increased apoptotic cells in the granular layer of the cerebellum.

Next, to test the integrity of the motor system, transcranial motor evoked potentials (TcMEP) were recorded. In this procedure, the motor cortex is provoked, and the signal propagated through the corticospinal tract is monitored by recording in a downstream muscle, such as the plantar muscle in the forelimb. The null mice had a significant reduction in the amplitudes of TcMEPs and prolonged onset latency. Surprisingly, although the mean amplitude of TcMEPs recorded in the heterozygous mice was comparable to WT mice, the TcMEP latency was prolonged almost to the same extent as in the null mice, which might point to a possible consequence of *Wwox* haploinsufficiency. Then, histological and transmission electron microscopy (TEM) assessment of the peripheral nervous system (PNS) through semi-thin transverse sciatic nerve sections revealed that even though the axonal organization and total nerve fibers were similar between experimental groups, the axons themselves were abnormally shaped and thinner with reduced axoplasm. This was accompanied by reduced endoneurium space, thinner myelin sheath, detachment of myelin lamellae, and increased apoptosis of Schwann cells (as seen by immunoelectron microscopy). 

This was followed by an examination of the CNS. Luxol fast blue (LFB) staining of myelin revealed reduced intensity in the commissural fibers (CC, hippocampal commissures), the association fibers (cingulum), the projection fibers emanating from the CC toward striatum, and the internal capsule, therefore affecting neuronal transmission tracts both inside a specific hemisphere, between hemispheres and from the cortex to the thalamus (both afferent and efferent). Furthermore, hypomyelination with atrophy of the optic tract and cerebellar foliar white matter was observed.

A noteworthy phenotype that was observed by this group was a middle interhemispheric fusion of the posterior frontal and parietal lobes, together with elongated roof plate and dorsal spinal cord malformation. This led to an examination of neuronal proliferation, migration, and differentiation. At E16.5, fewer proliferating (Ki67^+^) cells were seen in the neocortical subventricular zone (SVZ) and the cerebellum of *Wwox*-null mice. Additionally, reduced numbers of DCX^+^ cells, an early neuronal differentiation marker, were found when staining at E12.5, which supports the notion of reduced neurogenesis. This led to another dispute with the rat-derived data, when Chang et al., reported that the overall cortical thickness was significantly reduced in *Wwox*-KOs at E16.5 [50]. It is important to note that the SVZ is defined by the presence of intermediate progenitor (IP) cells, which give rise to most of the cortical neurons in the murine brain [51]. This stands in contrast to human neurogenesis, in which a major contribution arises from a histological structure called the outer SVZ (oSVZ) [52,53,54]. This structure is underdeveloped in rodents compared to humans.

Migration status was assessed using a pulse of bromodeoxyuridine (BrdU) labeling during embryonic development in utero at E16.5. In contrast to the localization of BrdU^+^ neurons to the cortical plate (CP) in the WT mice, increased numbers of BrdU^+^ neurons were found throughout the neocortex of *Wwox*-null mice, with a large portion still residing in the ventricular zone (VZ) and SVZ. Postnatally, ectopic neurons could be found in the cortex of *Wwox*-KO mice. Importantly, of the neurons in the VZ and SVZ, a larger portion in the null mice were Ki67^+^, which suggested that those neurons might be less differentiated, in addition to poor mobility during neocortical development [50]. At postnatal day 14 in the DG null mice, DCX expression was maintained for longer and NeuN expression was late to appear (day 20), suggesting a differentiation delay. These neuronal migration abnormalities during development and heterotopia were associated by the group with increased neuronal excitability, epilepsy, and mild to moderate mental retardation in humans and mice [50]. The epileptic aspect of the group’s research is expanded upon in another segment of this review.

Another observation was increased neuronal apoptosis in *Wwox*-null mice [50]. Together with the reduced proliferation in the SVZ discussed above, these findings are very important because high WWOX expression is usually associated with enhanced apoptosis [16,55,56], and loss of WWOX is associated with reduced check-point inhibition [12,13,15,57].

Finally, to dissect the role of different cellular populations in the brain, Repudi et al. used the conditional null mice previously reported [43], but utilized cell-type-specific expression of the Cre-recombinase, thereby generating the cell-targeted *Wwox*-KO mice. The targeted populations were neural stem cells (NSCs; *Nestin-Cre;* referred to as *N-KO*), neurons (*Synapsin-I-Cre; S-KO),* astrocytes (*GFAP-Cre; G-KO*), and oligodendrocytes and oligodendrocyte progenitors (*Olig2-Cre; O-KO*). Remarkably, at the reported time frame, only two models recapitulated the phenotype of the *Wwox*-null mice: the *N-KO*, in which WWOX was deleted in NSCs and its progenies, effectively knocking it out in all the cellular populations of the CNS. The second model was the *S-KO* in which WWOX is mutated only in mature neurons. It is important to emphasize that *N-KO* and *S-KO* recapitulated both neurological phenotypes (such as ataxia and seizures), but also systemic phenotypes such as growth retardation, metabolic abnormalities, and premature death by about 4-weeks of age. This effectively proved the major role of neuronal-WWOX in the phenotypes of the null mice.

Thereafter, our group proceeded to assess the conditional *Wwox*-null mice, *N-KO,* and *S-KO* mice and performed RNA-seq. This analysis revealed pronounced down-regulation of myelin and OLs-related genes. More specifically, genes involved in maturation (*Gjb1, Gjc2, and Olig1*) and myelin development, maintenance, and functionality of OLs (*Ermn, Ugt8a, Plp1, Otud7b, Mal, Eml1, Mobp, Hist1h2be, Cldn11, Mbp, Gal3st1, Fa2h, Gsn, Adamts4, Cnp, Mog, Oplalin, Enpp, Mag and Myrf*). This was in line with previous data arising from both rats [30] and mice [50], proving the significance of this phenotype. As the presence of changes in OLs in a model that specifically ablates WWOX in mature neurons (*S-KO*) could seem peculiar, we further evaluated populational changes by using both electron microscopy, immunofluorescence, LFB staining, RT-PCR, single-nucleus RNA-seq (snRNA-seq), and OLs-neuron coculture experiments. All the methods mentioned above led to the conclusion that ablation of neuronal-WWOX causes severe hypomyelination and decreased numbers of mature OLs in a noncell autonomous manner. Surprisingly, the numbers of OPCs were increased, which might suggest the problem lies in the maturation step between OPCs and mature OLs, a step in which the paracrine involvement of neurons has already been implicated [58,59,60]. These findings were recapitulated in both the cortex and hippocampus. 

Another important aspect of this study was the electrophysiological characterization. Local field potential (LFP) recordings in both live animals and brain slices demonstrated large amplitude bursting activity in *S-KO*, which was not present in the control, was localized to the superficial layer of the neocortex, and was attributed to hyper-excitability of the neocortical circuitry. Spectral power analysis showed increased power across many frequencies, such as the delta (< 5 Hz) and theta (5–9 Hz) rhythms, which have been implicated in epilepsy [61,62,63,64]. Another important finding was delayed signal conductance when stimulating in the CC and recording from neocortical layer V, which could be a result of hypomyelination. The connection between myelin abnormalities and susceptibility to seizures has been studied [65,66]. 

These revelations were expended upon by Breton and colleagues [67]. Recording from the neocortex of *S-KO* mice, spontaneous bursting was observed in both layer II/III and layer V; however, it was of larger amplitude in layer II/III as compared to layer V. Spectral analysis revealed the activity to be phase-amplitude cross-frequency coupled (PAC), corresponding to the coupling between theta (4–6 Hz) and high-frequency oscillations (100–400 Hz), as well as maximal coupling between delta (0.5–3 Hz) and gamma (30–90 Hz). By simultaneous recording from superficial and deep cortical layers and measuring the cross-correlation, propagation of the waveform was determined. More specifically, a low-frequency waveform (0.5–6 Hz) recorded in layer V lagged behind layer II/III, but not higher frequency oscillations, suggesting that the coherent activity of the low-frequency waveform predominantly propagated from superficial to deep cortical layers. Next, the generation of the spontaneous bursting events was studied using a whole-cell voltage clamp and selective pharmacological antagonists. Analyzing the reversal potential of pyramidal neurons supported the idea of excitatory currents dominance, probably glutamatergic. Accordingly, blocking glutamatergic neurotransmission with d-APV, a selective and competitive NMDA receptor antagonist, eliminated the spontaneous bursting event, with recovery after a washout step. In contrast, gap-junction blockade using CBX decreased the frequency and the duration of the bursting events, but not the amplitude, without recovery. Treatment with a specific gap-junction inhibitor, the pannexin blocker BB-FCF, did not recapitulate the effects of CBX, but did, however, shift the PAC from delta-gamma to delta-HFO ranges. Evaluation of miniature excitatory synaptic currents (mEPSCs) revealed increased amplitude but no significant changes to the frequency or decay time constant of mEPSCs (consistent with a postsynaptic mechanistic change), and with no overt change in the proportion of the NMDA: AMPA response. Monitoring the spontaneous excitatory and inhibitory postsynaptic currents (sEPSCs and sIPSCs) showed interesting results. While sEPSCs behaved similarly to mEPSCs and exhibited an increased probability of higher amplitude events but without changes in frequency, the sIPSCs were reduced in both amplitude and frequency for the *S-KO*s compared to controls, supporting a notion that *Wwox*-KO causes the neocortex to favor excitation over inhibition. Furthermore, injection of depolarizing current pulses into neocortical pyramidal neurons and interneurons was consistent with pyramidal neuron hyperexcitability but not in interneurons, which was supported by impaired membrane properties only in pyramidal neurons. Curiously, hippocampal slice preparations from control mice and *S-KO* mice were indistinguishable. One notable exception was when they stimulated in the stratum radiatum and recorded the population spike (PS) in the CA1 to CA3 direction; there was an elevated PS amplitude in the S-KOs as compared to WTs.

In conclusion, rodent models of *Wwox* loss-of-function can deeply enhance our understanding of neuropathology and the consequences of gene alterations. A wide variety of models exist, allowing research to evaluate these effects in both spontaneous mutations *Ide/Ide* rats, in global genetically edited knockdown (hypomorphic mice) and knockout (conventional and conditional null mice), and cell-type-specific *Wwox* knockout (cell-targeted KO mice). Although some differences arise between species and between models, the major phenotypes are recapitulated across groups and publications. Moreover, as some research highlights, the roles of WWOX in the context of cancer and tumor suppression are not always seen when studying the gene in the physiological, housekeeping context, as shown by the increased apoptosis and decreased proliferation reported by Cheng et al., [50]. This observation makes the study of the homeostatic role of WWOX in-vivo an absolute necessity. Altogether, these findings point to animal models as the gold standard for the study of WWOX-related brain disorders.

## 3. *WWOX* Loss-of-Function from the Human Perspective

### 3.1. WWOX in Human Neurodevelopmental Diseases

The discovery of the *WWOX* gene was made first in humans [10,18] and for many years its loss-of-function in humans was studied only in the context of its tumor suppression and adaptor protein functions [56,68,69,70,71,72,73,74,75,76].

In 2007, Gribaa et al. published a case report describing four patients from a consanguineous family from Saudi Arabia who suffered from early-onset generalized tonic-clonic epilepsy, ataxia, mental retardation, psychomotor delay, and learning disabilities, among other symptoms [77]. Importantly, the researchers were able to localize the source to the genomic locus 16q21-q23 through homozygosity mapping, but not to a specific gene. About 7 years later, Mallaret et al. published about another family, this time from an Israeli-Palestinian origin with two affected children, termed the disease as Spinocerebellar ataxia type 12 (SCAR12 syndrome) and, through studying both families, traced it back to the WWOX gene. Interestingly, both mutations were founder missense mutations (p.P47T and p.G372R). 

Shortly after, Abdel-Salam et al., (2014) has published the case of a consanguineous Egyptian family of which two individuals were affected by WWOX mutation [78]. The index patient suffered from a nonsense mutation (p.R54*) leading to neonatal growth retardation, microcephaly, optic atrophy, severe psychomotor delay, and intractable epileptic seizures that started as early as 2 months of age. The upper and lower extremities showed increased tendon reflexes and flexor plantar responses, which are associated with chronic central nervous system disease and upper motor neuron damage [79]. Ophthalmological examination indicated delayed visual maturation, bilateral severe macular and optic nerve dysfunction [78]. Cranial magnetic resonance imaging (MRI) demonstrated supratentorial and cortical atrophy, hypoplasia of the hippocampus, CC, and the temporal lobe with consecutively widened subarachnoid space. The older sister of this patient was reported to be born with similar symptoms and died prematurely by the age of 3 months. Some very important observations were made – first, in contrast to the rodent data, the metabolic screen appeared normal. Second, there was no history of cancer in the older generation of the reported family, although some carried heterozygous mutations in *WWOX* [78]. 

This report was soon followed by the publications of six additional patients from the United Arab Emirates and Portugal suffering from this developmental and epileptic encephalopathy (DEE) [80,81], which harbored either homozygous microdeletions, deletions, and insertions-deletions (indels) in the *WWOX* gene. At this point, Mignot et al. proposed the name WWOX-related epileptic encephalopathy, or WOREE syndrome [81] (now also termed DEE28). 

New cases are published every year from across the globe, expanding our knowledge regarding the disease’s features, such as affected populations, type of mutations, the defining symptomatology, and neurological abnormalities, and the age of onset, all of which have been extensively reviewed [2,82]. For example, Valduga et al. reported the first fetus with *WWOX*-null genotype, which presented with brain abnormalities on MRI and high rhythmic fetal movements [83], supporting the notion that *WWOX* germline mutations cause in humans neurodevelopmental disorders that start during embryonic development. Furthermore, most patients were reported with a compound heterozygous state [2,81,82,84,85,86], suggesting the disease can appear in non-consanguineous families. Finally, a few WOREE patients progressed into defined forms of epilepsy, such as West syndrome and Lennox–Gastaut syndrome, which expands its associated phenotypes [82,87]. 

Therefore, it is currently accepted to view SCAR12 and WOREE syndromes as a spectrum of disorders, with WOREE syndrome generally considered the more aggressive disease, associated with younger age of onset, more severe phenotype, and with premature death. Furthermore, it is associated with more extreme genetic changes, such as nonsense mutations, copy number variations (CNVs), and large-scale deletions [2]. This is in contrast to SCAR12, which is mainly associated with missense mutations [2,48]. Therefore, many claim a genotype-phenotype correlation [2,49,81,83,87]. 

The keen-eyed reader might notice that although the first description of neurodevelopmental consequences of *WWOX* mutations in humans preceded the mice research, data from the *Ide/Ide* rats “alerted” the medical field as early as 5 years before the first patient was described. Additionally, the first histopathological assessment was published only in 2020 [38], a period in time in which rodent data had already vastly accumulated. Therefore, one could say that *WWOX* research follows a “rodent to human” timeline.

### 3.2. The “Human Approach” to Disease Modeling of WWOX Loss of Function

#### 3.2.1. Rationale

Modeling WWOX loss of function in rodents advanced our knowledge considerably, as could be appreciated from the previous sections of this review. Despite this, the genetic background and brain development of a specific patient cannot be modeled in a mouse and, so far, rodent models have used global ablation of WWOX expression, and as a result, they do not discriminate between the two syndromes. Therefore, in our opinion, the experimental data obtained need to be validated in human tissues, and we view these validations as an absolute necessity in order to advance patient care. One of the main issues is the accessibility of human CNS tissue, which is limited in general, and samples from WWOX-related neurological syndromes are even rarer. In the following section of this paper, we review the human systems available for the study of WWOX neurodevelopmental disorders and the data they yielded. 

#### 3.2.2. Human Tissue

Early research regarding WWOX expression in human tissue revealed expression of WWOX in the adult human nervous system including the soma and dendrites of neurons residing in the cerebrum (frontal and occipital cortices, caudate nucleus, and limbic system, all of which contained also *WWOX*-expressing astrocytes), pons, medulla and all three layers of the cerebellum [23]. In the PNS, WWOX was seen in autonomic ganglia and Schwann cells [23]. Low to negative expression was also observed in the parietal cortex, temporal cortex, and the substantia nigra. This was one of the earliest indications of *WWOX* involvement in the human nervous system. 

The first and only paper to this date describing histopathological data from a WOREE patient came out in 2020 by Iacomino et al., [38]. The group studied the brain of a single fetus suffering from WOREE syndrome that was terminated at the 21st gestational week. MRI imaging found cerebellar vermis hypoplasia as the only macroscopic anatomical defect. Histologically, they reported abnormal formation of the developing cerebral cortex, anomalous migration of the external granular layer within the molecular layer (a phenotype that was validated also in *Ide/Ide* rats and WWOX knock-down NPCs [38]), disorganization of irregularly distributed glial trajectories and thin vascular network below in the external granular layer. 

Kosla et al., took advantage of the deposited next-generation sequencing (NGS) data by the RIKEN-led FANTOM5 consortium to determine *WWOX* gene expression levels in different human brain regions. Briefly, generated full-length cDNA clones were sequenced as single-molecules, and using Cap Analysis of Gene Expression (CAGE) their frequencies were monitored and transcription initiation events were mapped at a single base-pair resolution. The result was a database containing sets of transcripts, transcription factors, promoters and active enhancers of many mammalian primary cell types and cancer cell lines annotated to provide precise location [88]. This analysis found that the highest expression of *WWOX* in the adult is detected in the CC, medulla oblongata and cerebellum, and that the lowest levels are seen in the postcentral and paracentral gyrus. Moreover, adults display greater *WWOX* expression in the brain as a whole than 20–33 weeks fetuses, almost by 2-folds. 

In a 2020 review, Aldaz and Hussain [8] explored the Human Brain Transcriptome (HBT) and RNA-seq GTEx databases and found uniform transcript expression between brain regions from conception to adulthood in all depicted brain regions such as the cerebral and cerebellar cortices, hippocampus, amygdala, striatum, and mediodorsal nucleus of the thalamus. That being said, *WWOX* expression changes between developmental stages. In early embryonal life, expression is relatively high and then it slowly decreases during fetal development until birth. Postnatal *WWOX* expression increases gradually until adolescence and remains high. One possible explanation is a connection between cellular maturation in the CNS and *WWOX* expression. The authors highlighted the cerebellum for having the highest *WWOX* levels and the steepest increase in its expression throughout development. This contrasts with data from mouse brain taken from the Allen Brain Atlas that showed murine *Wwox* mRNA to be most expressed in the hippocampal formation and amygdala. *WWOX* expression in the human brain is summarized in Figure 1.

#### 3.2.3. Cell Lines

Human neural progenitor cells (hNPCs). Kosla et al. studied human neural stem cells (hNSC), derived from H9 embryonic stem cells (hESC) [89]. As these cells were unable to differentiate to OLs and astrocytes under spontaneous differentiation conditions, they were deemed hNPCs. *WWOX* was silenced using shRNA – resulting in WWOX-knockdown hNPCs. Functional assays found reduced mitochondrial redox activity (in line with data from research in mouse models outside the CNS [14,90,91]), stronger adhesion to fibronectin, and lowered pro-MMP2 and pro-MMP9 metalloproteinase secretion. When cultured in 3D, cells that underwent *WWOX* knockdown, in contrast to control hNPCs, ceased to proliferate and differentiate and did not form cellular networks as complex as in the control cells. 

This was followed by transcriptomic analysis by CAGE of both *WWOX*-knockdown hNPCs and *WWOX*-knockdown neurons (differentiated from the hNPCs). WWOX silencing caused differential expression of 2282 genes between control hNPC and *WWOX* knockdown hNPCs, and 7392 genes between the differentiated control neurons and *WWOX*-knockdown neurons. Gene ontology (GO) terms and gene-set enrichment analysis (GESA) identified these genes to be related to neural crest differentiation and migration, and to cell-cell adhesion present in the control hNPCs. Computing these results into the STRING database found that in neurons, these genes were related to neuronal migration, membrane proteins, cytoskeleton, and cell signaling. In the hNPCs, the most significant upregulated genes were related to signaling and chromatin remodeling. Finally, Principal component analysis (PCA) for all groups (hNPCs and neurons) implicated oxidative catabolism and oxidative stress genes as the main source for partitioning between *WWOX*-depleted and intact *WWOX* cells. 

Iacomino et al. re-analyzed this transcriptomic data, focusing on genes associated with neuronal migration and differentiation, and found reduced expression of some neural migration-related genes, such as microtubule proteins and kinesin family proteins [38]. 

Overall, these studies introduced two new human systems for the study of *WWOX* in the human nervous system, implicating it as important for biological processes such as mitochondrial redox, cell adhesion, and neuronal migration and differentiation. 

Human neuroblastoma. SH-SY5Y is a human neuroblastoma cell line that upon treatment with retinoic acid (RA) can give rise to differentiated human neuron-like cells [92], which makes it a good model to study neuronal differentiation. Wang et al. found that upon RA treatment, the differentiation of SH-SY5Y cells was associated with increased *WWOX* expression, together with decreased phosphorylation of the Tau protein at both Ser396 and Ser404 (phosphorylation at Ser422 was unchanged) [93]. The connection between Tau and WWOX has been long known [94] (as addressed later on in this review), but since Tau isoforms were suggested to be phosphorylated by GSK-3 *in-vivo* [95], and since GSK-3*β* has been studied in the context of neuronal differentiation [96], the group tested markers for its activity in response to RA treatment (levels of the inactive Ser9-phosphorylated GSK-3*β* and the phosphorylated *β*-catenin), which remained normal. A plausible explanation for the changes in Tau could be reduced GSK-3*β*-mediated phosphorylation, which together with the increased *WWOX* levels, suggested a connection between the two proteins. This could very well be supported by unrelated research that found that phosphorylation of GSK-3 proteins at serine residues is not the major regulatory mechanism in the CNS [96]. Furthermore, upon RNAi-mediated inhibition of *WWOX*, the SH-SY5Y cells displayed increased phospho-Tau at Ser396 levels and decreased neurite outgrowth [93]. Consistent with this observation, Tau hyperphosphorylation is increased in whole-brain tissues isolated from *Wwox*-null mice (unpublished data, Aqeilan Lab). Wang et al. further performed bioinformatic analysis and found putative GSK-3*β* binding motifs in WWOX, residing in the SDR domain (which contained an LXXRL motif similar to known binding partners of GSK-3*β*). The authors further validated this binding through immunofluorescence and immunoprecipitation experiments. Importantly, the interaction itself was not RA-dependent. *In-vitro* kinase assay allowed the researchers to demonstrate a negative regulation of GSK-3*β* by WWOX. Finally, the researchers found that this negative regulation promotes microtubular assembly, crucial for neurite growth, and that the mediator is the Tau protein. Overall, a molecular mechanism was suggested, in which upon RA administration, neuronal differentiation is induced, WWOX is upregulated which in turn inhibited GSK-3*β* and Tau phosphorylation, therefore promoting microtubule assembly and neuronal differentiation. 

Overall, modeling *WWOX* loss-of-function in cell lines allowed for in-depth analysis of the affected transcriptome and CNS-relevant molecular partners, but overall lacked the whole human tissue and complex 3D interactions between different cellular populations. Therefore, here enter brain organoids.

### 3.3. 3D Human Systems

#### 3.3.1. Introduction to Brain Organoids

In the passing two decades, two major breakthroughs have considerably enhanced our ability to generate a human system to study human development and disease: The first is the discovery of reprogramming of somatic cells into induced pluripotent stem cells (iPSCs) [97,98,99,100]. Human pluripotent stem cells (hPSCs), a group of cells generally composed of embryonic stem cells (ESCs) and iPSCs, are immortal cells that can be cultured and potentially used to generate every cell type in the petri dish [101]. Thus, they can be used to retain the specific genetic background and developmental stages that are unique to humans in general, and even a specific patient.

The second is the discovery that stem cells can self-organize to form a 3D culture that is highly similar, and sometimes identical to human tissue in terms of histology, developmental stages, functionality and cellular properties [102,103,104,105]. Therefore, generating organoids from hPSCs has the potential to model the complex physiology, cellular interactions, and disease pathology of a specific organ in a dish. 

More specifically, CNS maladies are challenging to study and treat due to the relative inaccessibility of functional human brain tissue [106,107]. This research field was revolutionized with the discovery that hPSCs have the intrinsic capability to generate cortical-like and cerebral-like tissues, which mimic much of the developmental stages, cellular polarity, and cell diversity seen in the developing embryonic CNS [108,109,110]. This model is attractive from a clinical stand-point due to the better generalizability of the data derived, the inherent species background, the cellular, mechanical, and topographical cues that are lacking in planar systems, and the ability to personally model CNS tissue in a dish (using patient-derived iPSCs) [111]. These *in-vitro* generated cortical tissues can be referred to as brain organoids and encompass a wide variety of protocols [112,113]. Traditionally, some used a “patterned” organoid generation approach, yielding a specific brain region, such as the cerebral cortex [108,110,114,115,116], hippocampus [117], cerebellum [118], and many additional CNS regions [115,119,120,121,122,123,124,125]. Others used an unpatterned approach to assume more “general” organoids, referred to here as cerebral organoids [109,126,127]. As the field progress, people find new and exciting ways to enhance the protocols – by fusing region-specific organoids, which are referred to by some as assembloids [123,128,129,130,131,132,133], bioengineering organoids [116,134,135], adding blood vessels [136,137], adding microglia [138,139,140], and even transplanting in a mouse brain [141].

However, brain organoids are not fault-proof. As could be expected from a new model that attempts to recapitulate brain development outside the uterus and in a dish, several works have raised concerns regarding its integrity. For example, the classic organoids quickly increase in size to a diameter that surpasses the diffusion limit, therefore the core becomes necrotic [109,115,127]. Furthermore, some concerns were raised regarding the cellular markers expression, correct maturation and regional identity, elevation of stress-related pathways (such as hypoxic stress), and the ability to achieve homogeneous, reproducible organoids [142,143,144,145,146]. Lastly, organoids recapitulate only embryonic brain development, with different time points resembling different gestational stages. For example, a type of brain organoids referred to as cortical spheroids transcriptionally resembled mid-fetal development (around 24 weeks post-conception) after 2.5 months of culture [110,146]. That being said, long-term cultures of the same organoids were reported to have a prominent post-natal transcriptional signature when cultured longer than 300 days, which was also confirmed with functional assays [147].

Despite this, organoids were used to study *in-vitro* many aspects of human brain development, such as myelination [129,148,149], astrocyte maturation [150], and cortical folding [151,152]. In particular, organoids are useful to study human NSCs, also called radial glia cells (RGs) [107,142]. These cells are generally divided into two distinct populations. The ventricular RGs (vRGs) that reside in the VZ, are derived from the neuroepithelium and are practically the origin for all neural cells in the CNS [51,52,53,54]. The other type is called outer RGs (oRGs), and can be considered a defining feature of the oSVZ, a major source of neurogenesis and gliogenesis in humans, and are rare in rodents but are abundant in brain organoids [52,53,54,107,142]. This way, the organoids recapitulate the formation of the human VZ-SVZ and cortical plate (CP) structure [109].

Moreover, organoids have been successfully used to model many brain pathologies [106,107]. These include but are not limited to viral infections (Zika, herpes simplex virus 1 and SARS-CoV-2) [115,153,154,155,156], brain tumors [157,158,159,160], microcephaly [109,154,161,162], neuropsychiatric disorders [163,164,165], ASD [166,167], neurodegenerative disorders (Alzheimer’s disease, Parkinson’s disease and frontotemporal dementia) [139,168,169,170,171,172], lissencephaly (Miller-Dieker syndrome; also associated with epilepsy) [173], and seizure-related disorders [174] such as Tuberous sclerosis [175], Angelman syndrome [176], Rett syndrome [177], Timothy syndrome [128], and developmental and epileptic encephalopathies - *UGDH* gene mutations (DEE84) [178], SCAR12 and WOREE syndromes [44,57].

#### 3.3.2. WWOX in Brain Organoids

As organoids can be a powerful tool in the study of human disease, our group sought to apply them to WWOX mutations. To do that, we generated *WWOX*-KO hESCs from the WiBR3 cell line. These cells were then used to generate *WWOX*-KO cerebral organoids (COs) [57]. After validating the organoids, the first step was to find out if the organoids can recapitulate hallmarks of epilepsy, such as neuronal hyperexcitability and gliosis [33,34,35,37]. To address the first, we recorded local field potential (LFP) from COs slices and observed increased firing in *WWOX*-KO COs. At baseline conditions, *WWOX*-KO COs displayed a spectral power (quantified by the area under the curve; AUC) approximately twice as high as in the WT COs. Upon administration of the convulsant 4-Aminopyridine (4-AP), the neuronal firing rate increased in all lines of COs, with the *WWOX*-KO COs being more reactive. Notably, this increased activity was observed in the lower frequency ranges, a phenomenon previously described in epilepsy [64]. Using cross-frequency coupling of the sample traces for both WT and KO lines in the presence of 4-AP revealed an increase in the *δ*: HFO frequency pairs—a signature that is associated with seizure sub-states [63]. These unforeseen results suggest that WWOX loss of function could result in neuronal hyperexcitability in the early stages of development. An observation we found interesting was a decrease in the oscillatory power of the COs during organoids maturation, which, though it fits previous reports in organoids [179], was more pronounced in the KO lines, potentially suggesting a maturation delay. 

The second hallmark, gliosis, which was also previously described in *Wwox*-null mice [49] but not in rats [30], was addressed by quantifying the expression of the astrocytic markers GFAP and S100*β*, both at protein and RNA levels, with additional markers (AQP4 and ALDH1A1) also quantified at the RNA level. Overall, WWOX-KO organoids demonstrated increased astrogenesis, which progressed with time [57]. 

We also examined *WWOX*-related phenotypes. First, we stained for *WWOX* and were surprised to discover that during the early stages of organoids culture *WWOX* expression was limited to the vRGs. Although unknown, this fits the description of the limited expression of *WWOX* mRNA during human embryonic development. This connection was further supported when upon prolonged culturing *WWOX* expression appeared in non-RG cells [57], and in another organoid model of ours, was even found in neurons [44]. This pattern is highlighted in Figure 1. 

Next, we checked if these organoids could recapitulate the myelin-related phenotype reported in mice and rats. Since COs lack OLs, we switched to an organoid protocol that is enriched with it, the oligocortical spheroids (OS) [148], and generated the *OS-WWOX-KO* system [44]. These spheroids recapitulate many of the phenotypes of the *WWOX-KO* COs, especially the electrophysiological phenotype, and demonstrated apparent hypomyelination, validated through immunofluorescent staining, qPCR, and electron microscopy. 

As described so far, *WWOX*-*KO* organoids shared many common features with the rodent models, but this was not always the case. A major difference was seen in the inhibitory neuron population. In contrast to the decreased levels of GABAergic neurons in *Wwox* null mice [49], we found increased GABAergic markers expression, which made us hypothesize that there is a role for the developmental depolarizing GABA currents in the pathogenesis [57].

As a functional assay for *WWOX*, the COs system enabled us to explore the physiological DNA damage response (DDR), a well-known function linked with proper *WWOX* expression [13,15]. As WWOX was mainly found in the vRGs, we zoomed into the VZ to study the DDR. Immunostaining of *γ*H2AX and 53BP1, surrogate markers of DNA breaks, revealed accumulation of damage foci in the VZ in *WWOX*-KO COs, suggesting impaired DDR. In addition, staining for the proliferation marker Ki67 revealed an increased number of proliferating cells harboring *γ*H2AX foci, suggesting loss of checkpoint inhibition, another previously described function of WWOX [13,15]. This was also accompanied by diminished apoptosis in the vRGs, which altogether fits the notion of prolonged survival of damaged vRGs. Notably, studies in human and mouse NSCs found that accumulation of DNA damage foci, either in the nuclear or mitochondrial DNA, can cause the NSCs to stop differentiating into neurons and to assume an astrocytic-like phenotype [180,181], which in part can explain the increased astrogenesis. 

Next, to unbiasedly study unknown molecular changes, we performed bulk RNA-seq. GESA and GO terms analysis revealed many affected pathways, such as processes related to ATP synthesis coupled electron transport, oxidative phosphorylation, glycolysis and gluconeogenesis, and negative regulation of cell cycle. All are consistent with known functions of WWOX from other models [13,14,15,73,90,91]. Additionally, enrichment was seen in genes related to neuron fate commitment and specification, supported by the work in neuroblastoma cell lines that connected *WWOX* to GSK-3*β* and neuronal differentiation [93]. This observation was interesting, as the most enriched pathways were related to ‘regionalization’ and ‘axis specification’ (Ventral-Dorsal & Anterior-Posterior), which after closer inspection, included many genes connected to the Wnt-pathway. Since WWOX has been implicated in this pathway [22,50,76,93,182], we pursued this lead and found chronic Wnt-pathway activation in our *WWOX*-KO COs that is not seen in the control organoids. The observation was followed by the finding of disrupted cortical layers, a previously reported consequence of Wnt activation in brain organoids [134], and a finding that was seen in a fetus suffering from WOREE-syndrome and in *Ide/Ide* rats [38]. This to us was reminiscent of cortical dysplasia, which has a well-recognized role in the pathogenesis of drug-resistant epilepsy [183,184,185].

Lastly, we generated WOREE syndrome COs. These COs were obtained by reprogramming somatic cells from a WOREE patient and his healthy heterozygous parents, resulting in WOREE syndrome iPSCs. This model validated most of the described phenotypes in *WWOX*-KO COs [57]. Notably, cell-attached recordings from these organoids documented a four-fold increase in the neuronal firing rate, a ratio that was observed also in our *Wwox*-null mice [186]. Overall, WOREE syndrome COs achieved two important goals: The first was validating the applicability of the *WWOX*-KO COs to human patients, and the second, by recapitulating much of the rodent data, this model validated it as well. 

As most of the phenotypes were observed by us in the cortical parts of the organoids, forebrain-specific organoids (FOs) were generated [57,115]. WOREE-syndrome FOs recapitulated and validated the phenotypes observed in the COs. Reprogramming somatic cells from a SCAR12 syndrome family and generating the SCAR12 syndrome iPSCs and SCAR12 syndrome FOs highlighted some differences with WOREE syndrome [57]. In fact, SCAR12 syndrome FOs lacked most of the histopathological phenotypes seen in WOREE syndrome organoids, except for the Wnt-activation signature. Therefore, this proves the ability of the organoid system to differentiate between the diseases and might be the only available SCAR12-specific model to date. 

Work by Gordon et al., which investigated cortical spheroids development in long-term culture compared to human brain tissue, described developmental trajectories for gene expression and highlighted in them a few neurological diseases-related genes [147]. One of the genes found to have differential expression during development in the epilepsy cluster was *WWOX*, which was expressed early in prenatal development, was downregulated, and reappeared later in post-natal developmental stages (long-term culture of cortical spheroids). 

Another interesting work was done by Esk et al. The group developed a new gene screening tool based on cerebral organoids called CRISPR-LICHT, which they applied to study microcephaly-related genes [187]. For sake of the simplicity of the explanation, the system is based on the infection of hPSCs with a single-guide RNA (sgRNA) library, multiple barcoding system, and single-cell next-generation sequencing to perform lineage tracing in the organoids. Roughly, the idea is that after induction of CRISPR-editing, the lineage in which the affected gene is related to microcephaly will be smaller in relation to its size if CRISPR-editing was not induced. One of the top candidate genes they found was *WWOX,* whose mutated lineage was reduced at day 42 of culture following CRISPR induction. Microcephaly was not seen in our size evaluation [57]; therefore this observation is very important and worthy of further studies in light of a review describing microcephaly in only about 30% of the patients [82], and a genome-wide association study (GWAS) that found an intronic single nucleotide variant (SNP) in *WWOX* (rs10514437) to be a possible influencer of infant white matter volume, but only approached genome-wide statistical significance [188].

In total, the use of brain organoids, in our opinion, can be an important model for the study of *WWOX* in the CNS, combining both known phenotypes from primary human tissues and rodent models, and the specific genetic background of humans in general, and patients in particular. 

## 4. Other *WWOX*-Related Neurodevelopmental and Neurodegenerative Disorders

### 4.1. WWOX and Autism Spectrum Disorder

Autism spectrum disorder (ASD) is a neurodevelopmental disorder characterized by the key features of impairment of social communication and interactions, accompanied by stereotyped and repetitive patterns of behavior, interests, and activities [189]. ASD is considered to have a genetic, epigenetic, and environmental background. Usually, it is associated with copy number variations (CNVs) which contribute at about 15% to the causes of ASD, and single nucleotide variations (SNVs) which contribute at 7%. Only a few genetic alterations have such complete penetrance that they are associated with ASD in almost every person who carries that variant [190].

*WWOX* was first reported to be involved in ASD in 2016, through an analysis of the Autism Genetic Resource Exchange (AGRE) database combined with the Simons Simplex Collection (SSC) dataset, which found CNVs in 12 affected children (but only one unaffected) involving the *WWOX* gene, suggesting it to be a low-penetrance ASD-locus [191]. However, the chr16q23.1 region, which contains *WWOX,* was associated with ASD as early as 2011 [8]. Since then, compound heterozygous mutations were implicated in a case report [192] and other populations-based studies [193,194]. For example, Bartnik et al., (2012) reported a patient with an isolated deletion that was associated with ASD and epilepsy [195]. 

Similarly, *WWOX* was associated with intellectual disability (ID) and attention deficit hyperactivity disorder (ADHD) [8]. Overall, it seems CNVs in the *WWOX* gene can result in other forms of neurodevelopmental disorders besides WOREE and SCAR12 syndromes. Understanding what determines which phenotype develops is a source of great interest. At present, there are no dedicated models to study WWOX function in ASD, ADHD, and ID. 

### 4.2. WWOX and Alzheimer’s Disease

The association between WWOX and neurodegeneration has been studied for a long time but did not follow the “rodent-to-human” timeline that was described so far for the encephalopathies. As it has been extensively reviewed elsewhere [4,6,7,9], and for the sake of clarity, it is briefly described here with the main focus being the different models used through the years between studies and research groups. 

Alzheimer’s disease (AD) is a heterogenous neurodegenerative disease and is the most common cause of dementia in the elderly [196]. The brain atrophy associated with AD is progressive, with different patterns of spread. For example, in late-onset AD, the atrophy is first seen in the medial temporal regions before spreading to other regions of the neocortex, such as the parietal, occipital, and frontal cortices [197]. The brain atrophy in the entorhinal cortex is generally in correlation to the onset of cognitive impairment [196]. At the molecular level, there is a development of dystrophic neuritic plaques containing amyloid-beta (A*β*) and neurofibrillary tangles (NFTs; composed of hyperphosphorylated tau filaments), microglia and astrocytes activation, and synaptic and neuronal loss [196,198]. Additionally, A*β* accumulates in blood vessel walls in the cortex and leptomeninges [199].

The story of *WWOX* and AD began in 2004 when Sze et al. examined the hippocampi of Alzheimer’s disease patients and found significant down-regulation of the WWOX protein (also in phosphorylated form), while phosphorylation of Tau and NFT formation were significantly up-regulated [94]. Low levels of WWOX were observed also in the dystrophic neuritic plaques. Furthermore, in neurons from AD hippocampi, Tau colocalized with endogenous WWOX, which suggested physical interaction. This was followed by knockdown of *WWOX* in the human neuroblastoma cell line (SK-N-SH cells) using small interfering RNA (siRNA). This manipulation increased Tau phosphorylation in sites associated with enhanced ERK and GSK-3*β* activity and increased NTFs. Interestingly, in *WWOX* knockdown mouse fibroblasts (*siWWOX* transfected L929 cells) exposure of these cells to a MEK1/2 inhibitor (PD-98059) and a JNK inhibitor (SP600125) inhibited NFTs. Lastly, *WWOX* was shown to physically interact with Tau and GSK-3*β*, an interaction that was enhanced by Estradiol (E_2_) treatment. They were also able to map the interaction between *WWOX* and Tau to the SDR domain. As mentioned before, later on, research in SH-SY5Y neuroblastoma cells showed that *WWOX* physically interacts with and inhibits GSK-3*β*, preventing Tau hyperphosphorylation [93]. 

Another important discovery was the accumulation of trafficking protein particle complex 6A (TPC6A*),* its isoform TPC6AΔ, and Tau aggregates in the brain of conditional *Wwox*-null mice [200]. TPC6AΔ protein is related to caspase activation, Tau aggregation, and A*β* generation in patients with Alzheimer’s disease [200]. Additionally, *WWOX* knockdown using siRNA in COS7 cells (African green monkey kidney fibroblast-like cell line) caused aggregation of ectopic TPC6AΔ and TGF-*β*1-induced antiapoptotic factor (TIAF1). This report followed a previous paper that implicated TGF-*β* signaling, TIAF1 self-aggregation, and nuclear accumulation of SMAD4 protein in suppression of amyloid precursor protein (APP) phosphorylation and induction of A*β* [201]. TIAF1 aggregates were also found in AD hippocampi [201]. This pathway is of particular interest, as WWOX was also associated with SMAD4 translocation to the nucleus [29]. Further studies in cell lines and *WWOX*-KO MEF cells isolated from the conditional *Wwox*-null mice found that *WWOX* physically binds TPC6A and TPC6AΔ, and prevents its aggregation [201]. These studies led to a model in which *WWOX* dysfunction causes TPC6AΔ polymerization leading to the aggregation of TIAF1 and caspase activation that causes APP degradation, leading to the generation of A*β* and the formation of the NFTs, causing neurodegeneration. 

In addition, in a triple-transgenic mice model for AD, treatment with Zinc finger-like protein that regulates apoptosis (Zfra) peptides blocked tau aggregation and Aβ formation and restored memory deficits [202]. Then, the research group used another model in which injection of melanoma B16F10 cells to nude mice cause neurodegeneration. Zfra treatment reduced the percentage of apoptotic nuclei in the hippocampus and specifically suppressed WWOX phosphorylation at Ser14, which is a form of *WWOX* they associated with AD progression and severity [202]. Notably, they used in this study also *WWOX* heterozygous mice and observed faster age-related decline in both short- and long-term memories than those in the triple-transgenic mice model.

Finally, in 2019, a massive GWAS meta-analysis study named the *WWOX* gene as a risk locus for late-onset AD [203].

To conclude, *WWOX* seems to be connected to neurodegeneration. Although these are diseases of the adult, cell lines, *Wwox*-null mice and *Wwox* heterozygous mice can serve as good models for AD and cognitive decline. Furthermore, as organoids have been used to study both *WWOX* mutations in neurodevelopment [44,57] and neurodegeneration [139,168,169,170,171,172], it is exciting to speculate whether this model can also be applied to study *WWOX* in diseases such as Alzheimer’s.

### 4.3. WWOX and Parkinson’s Disease

Parkinson’s disease (PD) is a progressive neurodegenerative disorder and the second most common age-related neurodegenerative disease, exceeded only by AD [204,205]. The disease can manifest with motor and nonmotor symptoms, but the cardinal features of the disease typically include rest tremor, rigidity, bradykinesia, and gait dysfunction with postural instability [205]. In May 2021, a longitudinal genome-wide survival study (GWSS) analyzed whole-genome sequencing data from 4491 samples originating in PD patients and found alternations in the *WWOX* locus to be associated with a greater risk for PD progression into Parkinson’s disease dementia (PDD) and cognitive deterioration [206]. A possible connection between *WWOX* and PD was shown 13 years earlier by studying the effects of neurotoxin-induced PD on *WWOX* [207]. In this study, the researchers used a model for PD in which either rats or human neuroblastoma cell lines were treated with 1-methyl-4-phenyl-pyridinium ion (MPP^+^). MPP^+^ is a product of the drug 1-methyl-4-phenyl-1,2,3,6-tetrahydropyridine (MPTP), which, after crossing the blood-brain barrier (BBB), is metabolized and MPP+ is concentrated into dopaminergic neurons via the dopamine transporter, causing neurodegeneration. This damages the basal ganglia and the substantia nigra, causing parkinsonism [208]. The researchers observed that MPP^+^ injection into the corpus striatum (CS) caused suppression of WWOX expression in nerve bundles but upregulated it in apoptotic neurons postinjury for 1–3 days, both in the CS and in the ipsilateral cortex [207]. Interestingly, WWOX accumulated also in the nuclei of the degraded neurons and persisted for several weeks. Additionally, WWOX could be observed in the nucleoli, mitochondria, and myelin sheath. WWOX was shown to interact with JNK1 in both MPP^+^-treated neurons and neuroblastoma SK-N-SH cells. MPP^+^ treatment in this cell line caused phosphorylation of both and reduced the interaction. In rat cortical lysates treatment caused increased binding with JNK1 in the cortex both in the contralateral and ipsilateral sides 1-h post-injection, followed by dissociation at 4-h, with variable results when examining the ipsi and contralateral CS. This interaction of WWOX and JNK1 could be of interest, as WWOX and JNK1 were shown to counteract each other and to inhibit WWOX-mediated apoptosis [56,207]. This idea was supported by the observation that inhibition of WWOX activation attenuated MPP^+^-induced reduction in cell sizes that the authors linked to apoptosis. 

Notably, another CFS gene, *PARK2* (Parkin) was found to be involved in autosomal recessive juvenile Parkinson’s disease (ARJP) [209,210]. Overall, although the data connecting *WWOX* and PD is scarce, and future research might further support its role in the disease mechanism. 

### 4.4. WWOX and Multiple Sclerosis

As reviewed here, the connection between *WWOX* and myelin has been studied in several models, from rats to mice and brain organoids. All of these are relatively “young models”. The rodent models are short-lived while the organoids mainly model embryonic development. However, Multiple sclerosis (MS) is typically a disease of young adults, with an age of onset usually around 20 to 40 years of age, with only about 3–5% of patients reporting symptoms onset before the age of 18, with an incidence rate of up to 0.1 per 100,000 for individuals at an age <10 years, which is then referred to as pediatric-onset MS (POMS) [211,212]. 

MS is an autoimmune neurodegenerative disease characterized by chronic demyelination, inflammation, gliosis (plaques or scarring), and neuronal loss [211,213]. The clinical course is variable, with neurologic events usually following either a relapsing-remitting or a progressive course, and can range from relatively benign to a rapidly evolving and incapacitating disease [211,213]. MS plaques are typically disseminated in time and space (developing at different time periods and in different CNS locations). 

*WWOX* was first implicated in MS through a GWAS that identified it as one of 48 new susceptibility loci [214]. Curiously, the *WWOX* variant was intronic (rs12149527). This finding was supported by the work of Jäkel et al., who performed snRNA-seq of postmortem white matter areas of MS patients’ brains and unaffected controls. In short, they identified subclusters of OLs in control human white matter and found that some subclusters were underrepresented in MS tissue, whereas others were more prevalent, suggesting different functional states of OLs in MS lesions. *WWOX*-expressing cells were identified as being reduced in chronic active MS lesions [215], which are defined by accumulation of microglia and/or macrophages at the lesion edge, subtle opening of the blood-brain barrier, and repair/remyelination failure with axonal loss, and are associated with more aggressive MS [216]. 

A recent review suggested mechanisms for *WWOX* involvement in myelin production [8]; one, through involvement in cellular lipid homeostasis [217] and the second, direct interaction with proteins involved in protein trafficking, endosome, and lysosomes networks, such as SIMPLE, a protein that was implicated in the autosomal dominant demyelinating form of the Charcot-Marie-Tooth (CMT) disease linked to 16p (CMT1C) [8].

Overall, it is becoming apparent that *WWOX* has an important role in myelination, and could be involved through multiple cells lineages, such as neurons (implicated by Repudi et al., [44]) and oligodendrocytes (as observed by Jäkel et al., [215]). The mechanism is currently still unknown; therefore, making further research and developing dedicated models is warranted. 

## 5. From the Bench to the Patient—How Can We Help?

Although an important goal of medical research is the understanding of disease processes, arguably equally important is improving medical care and the development of state-of-the-art approaches to treatment. Possible approaches would be using relevant models to find better treatments among already existing drugs, while others could be the development of new therapies. Several papers utilized WWOX models to do just that, and are discussed here.

An example of the use of experimental models to find therapeutic options from already approved drugs is seen in the research of Cheng et al. [50]. Here the model was used to study the epileptic phenotype by intraperitoneal injections of the convulsant drugs pilocarpine (muscarinic receptor agonist) and PTZ (GABA receptor antagonist). The *Wwox*-null mice showed higher susceptibility to the drugs, with about half progressing into *status epilepticus,* a phenomenon not observed in the WT or heterozygous mice. Interestingly, the null mice also were the only group that responded to pretreatment with the anti-epileptic drug ethosuximide, which suppressed PTZ-induced seizures. The researchers further combined the knowledge of the known interaction between GSK-3*β* and WWOX [93], and after they confirmed over-activation of GSK-3*β* (dephosphorylation at Ser9) they used pre-treatment with lithium, a drug which is highly used for bipolar disorders [218] that also inhibits GSK-3*β*, to suppress PTZ-induced seizures.

Next, in our mentioned above organoids work by Steinberg et al., we re-introduced the *WWOX* coding sequence into the safe-harbor *AAVS1* locus in *WWOX-KO* hESCs and WOREE syndrome iPSCs [57]. The resulting COs expressed *WWOX* diffusely in all cell types throughout the culture period and were rescued from most of the mentioned phenotypes, including the complete rescue of increased astrogliosis, cortical dysplasia, DNA damage, and neuronal hyperexcitability, and to some extent the Wnt-pathway activation. Furthermore, lentiviral transduction of *WWOX* into mature *WWOX-KO COs* was able to reverse the electrophysiological findings. That being said, the phenotype was not rescued completely and included the uncontrolled expression of WWOX in all cellular populations, which made us speculate that a cell-type-specific approach would be more appropriate. 

Therefore, in an article, by Repudi et al., a novel gene-therapy approach is presented [186]. This approach includes the generation of an adeno-associated viral vector serotype 9 (AAV9) vector, which ectopically expresses either mouse or human WWOX under the *Synapsin-I* promotor, i.e., specifically expressing in mature neurons. AAV vectors have been used clinically in several diseases [219,220,221], and AAV9 has specifically high CNS tropism [221,222,223,224]. The combination of AAV9 and the *Synapsin-I* promotor resulted in the highly selective expression of *WWOX* in neurons. Injection of *AAV9-hSynI-WWOX* to neonatal *Wwox*-null mice rescued the CNS phenotypes, including neuronal hyperexcitability, seizures, myelination deficits, and behavioral changes (anxiety and motor functions), results that were not seen in the *AAV9-hSynI-EGFP*-injected mice [186]. Furthermore, CNS neuronal-specific restoration of WWOX also rescued abnormalities in other tissues (bone and testis), as well as systemic symptoms such as hypoglycemia, growth retardation, and premature lethality. The success of this treatment both in and outside the CNS, together with the improvement in survival (i.e., successfully injected mice could be identical to wild-type mice), suggests that AAV9-delivery combined with neuron-specific expression of WWOX could serve as an efficient and safe treatment for *WWOX*-related neurodevelopmental disorders. 

All in all, and although much work is yet to be done, *WWOX* models have the potential to benefit human patients and should serve as a preclinical platform for testing and development of treatment. In the future, one may speculate that the generation of patient-specific organoids or mutation-specific rodent models could be utilized as a platform for the development of personalized medicine, drug screening, and safety enhancement. 

## 6. Concluding Remarks and Future Perspective

**Figure 2:**
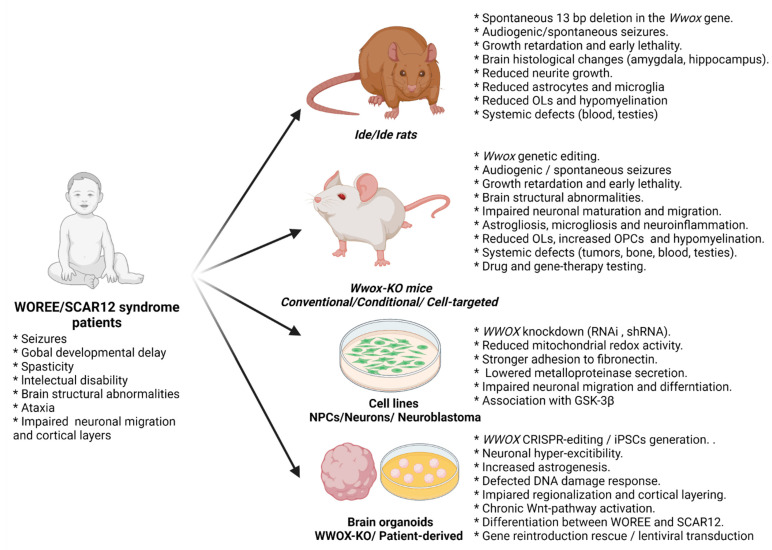
Summary of the available systems for modeling WWOX loss of function and the observed phenotypes.

Experimental models for WWOX-related CNS disorders existed even before the first connection between WWOX and neurological diseases was made. As the clinical and biological research progressed, enhancing each other, more complicated and precise models appeared with many researchers and laboratories contributing to the scientific effort. These model systems, animal or human, all have in common the recapitulation of major aspects of the neuropathology (Table 1, Figure 2). Despite this, no model is currently perfect and major drawbacks can be found in all of them. Therefore, we believe the combination of these models can benefit both the scientific community in the mechanistic understanding of the disease, and patients in the development of new and safe treatments.

## Figures and Tables

**Table 1:** Summary of the main models for studying WWOX-related neurodevelopmental disorders.

Heading	*Ide/Ide* Rats	*Wwox*-null Mice (Conventional, Conditional)	Cell-Targeted *Wwox*-KO Mice (Synapsin-cre (S), Nestin-cre (N))	Human Cell Lines (hNPCs, Neuroblastoma)	Brain Organoids (Cerebral Organoids, Forebrain Organoids, Oligocortical Spheroids)
Source of WWOX mutation	Spontaneous; experimental inbreeding	Genetic editing Targeting exons 1 or 2–4	Genetic editing targeting exon 1 in specific cell types	*WWOX*-knockdown (shRNA, RNAi)	Germline mutations (iPSCs) Genetic editing (CRISPR/Cas9)
Differentiation between WOREE/SCAR12	No	No	No	No	Yes
Brain structural changes	Extracellular vacuoles in the amygdala and hippocampus Neuronal layers migration defects Normal cortical thickness	Fused vermian lobules and foliation defects (cerebellum) Interhemispheric fusion of the cerebral lobes Elongated roof plate Dorsal spinal cord malformation Neuronal migration defects and heterotopia Reduced cortical thickness	Similar to *Wwox*-null mice *(N-KO* more than *S-KO)*	No	Cortical dysplasia Microcephaly (?)
CNS manifestations	Increased relative brain mass Ataxic gait Epileptic seizures Reduced neurite growth Reduced OLs and hypomyelination Reduced astrocytes Reduced microglia	Ataxic gait Epileptic seizures Reduced OLs and hypomyelination (CNS, PNS) Reduced interneurons subtypes (hippocampus) Loss of Purkinje cells (cerebellum) Impaired maturation and migration of neurons Reactive gliosis Increased TcMEP latency	Similar to *Wwox*-null mice *(N-KO* more than *S-KO)*	No	Neuronal hyperexcitability Enhanced astrogenesis/astrogliosis Reduced mature OLs and hypomyelination
Systemic symptoms	Dwarfism, decreased levels of plasma growth hormone Testicular and steroidogenesis abnormalities Blood biochemistry abnormalities Early post-natal lethality	Growth retardation Blood biochemistry abnormalities Bone metabolic defects High tumor burden Hematologic defects Testicular and steroidogenesis abnormalities Early post-natal lethality	Similar to *Wwox*-null mice *(N*-*KO* more than *S-KO)*	No	No
Molecular changes	-	CNS inflammation	CNS inflammation	Impaired neuronal migration defects	Impaired DNA damage response Chronic Wnt-pathway activation
Recapitulates developmental milestones	Yes	Yes	Yes	No	Yes
Modeling of human physiology	Yes	Yes	Yes	No	Partially; lacks some cell types, anatomical structures, and organ-organ interactions. Can be overcome by protocol adaptations.
Retains human genetic background	No	No	No	Yes	Yes Can be patient-specific (iPSCs)
Ease of manipulations/treatment	Difficult	Difficult	Difficult	Relative ease	Moderate ease
Cell-type dissection	No	No	Yes, inherent	Yes	Possible
Treatment development	No	Suppressed seizures with lithium treatment	Complete rescue using neuron-specific *WWOX* restoration (*AAV9-hSynI-Wwox)*	No	Partial rescue with diffuse *WWOX* restoration into hPSCs (AAVS1-WWOX) Neuronal hyperexcitability suppressed with Lenti-WWOX infection
Maintenance ease, cost	High maintenance, high cost	High maintenance, high cost	High maintenance, high cost	Low maintenance, low cost	Moderate maintenance, high cost
